# Food shaped photosynthesis: Photophysiology of the sea slug *Elysia viridis* fed with two alternative chloroplast donors

**DOI:** 10.12688/openreseurope.16162.1

**Published:** 2023-06-28

**Authors:** Luca Morelli, Paulo Cartaxana, Sónia Cruz

**Affiliations:** 1 CESAM – Centre for Environmental and Marine Studies, Department of Biology, University of Aveiro, Aveiro, Aveiro District, 3810-193, Portugal

**Keywords:** Kleptoplasty, NPQ, photoprotection, Sacoglossa, xanthophyll cycle.

## Abstract

**Background:** Some Sacoglossa sea slugs integrate intracellular chloroplasts derived from the algae they feed on, a process termed kleptoplasty. The stolen chloroplasts – kleptoplasts – can maintain their functionality up to several months and support animal metabolism. However, chloroplast longevity can vary depending on sea slug species and algal donor. In this study, we focus our attention on *Elysia viridis*, a polyphagous species that is mostly found associated to the macroalga *Codium tomentosum*,but that was reported to eat other macroalgae, including *Chaetomorpha* sp.

**Methods:** We have investigated the changes in *E. viridis* physiology when provided with the two different food sources to evaluate to which extent the photosynthetic and photoprotective mechanisms of the algae chloroplasts matched those of the plastids once in the animal cells. To perform the study, we rely on the evaluation of chlorophyll *a* variable fluorescence to study the photophysiologic state of the integrated kleptoplasts and the high-performance liquid chromatography (HPLC) to study variations in the photosynthetic pigments.

**Results:** We observed that the photosynthetic efficiency of *E. viridis* specimens is similar to the one of the respective algal donor but specimens fed with *Chaetomorpha* tend to have lower performances. Significant differences appear in the non-photochemical quenching (NPQ) abilities. While sea slugs fed with *C. tomentosum* react to high-light stress as the algae, *E. viridis* hosting *Chaetomorpha* chloroplasts are unable to properly recover from photoinhibition or perform a functional xanthophyll cycle (XC).

**Conclusions:** Our results showed that, even if the sea slugs fed with the two algae show photosynthetic activities like the respective algal donors, not all the photoprotective mechanisms present in *Chaetomorpha* can be maintained in *E. viridis*. This indicates that the functionality of the kleptoplasts does not depend solely on their origin but also on the degree of compatibility with the animal species integrating them.

## Plain language summary

Sea slugs known as Sacoglossa (also called "solar-powered sea slugs") have a fascinating ability to steal and use chloroplasts from the algae they eat. This process is called kleptoplasty. These stolen chloroplasts, also called kleptoplasts, can remain functional for several months and help the sea slugs with their metabolism by performing photosynthesis similar to plants. However, the time of chloroplast maintenance can vary depending on the species of sea slug and the type of algae they feed on. In this study, we focused on a species called *Elysia viridis*, which eats various types of algae, including *Codium tomentosum* and *Chaetomorpha* sp. These two algae have different characteristics when it comes to photosynthesis and protection against excessive light. We investigated how the physiology of *E. viridis* changed when it was given these two different food sources. Our results showed that while the sea slugs that fed on both types of algae had similar photosynthetic activities to their respective food, not all the protective mechanisms of Chaetomorpha algae could be maintained in *E. viridis*. This suggests that the functionality of the stolen chloroplasts depends not only on their source but also on how well they match the sea slug species that incorporate them.

## Introduction

Photosynthesis is a process used by phototrophic organisms to harness chemical energy from light. Generally associated to plants and algae, photosynthesis may occur in metazoans that establish endosymbiotic associations with algae. A particular case is that of Sacoglossa sea slugs that feed on macroalgae and maintain functional chloroplasts in their cells, a process termed kleptoplasty ( Cruz & Cartaxana, 2022). After having pierced the algal cell wall with its radula, the animal sucks and digests all the cellular components, including the nucleus, while the chloroplasts are maintained intact and are integrated in the animal cells. In some species, the stolen chloroplasts – kleptoplasts – can continue to function for several weeks to a few months, despite the absence of algal nuclear genes necessary to encode most chloroplast proteins. The process is often considered an example of endosymbiosis even if the chloroplast itself does not constitute an organism ( Dorrell & Howe, 2012).

The presence of kleptoplasts in the cells of the digestive tubules, which ramify throughout most of the sea slug’s body, contributes to the establishment of a colour resembling that of its algal food and helps the animal to blend with the environment to escape predators ( Hirose, 2005). Most likely, kleptoplasty established originally as a mechanism of crypsis as some species, such as *Placida dendritica*, can assume a green coloration by storing only non-functional kleptoplasts ( Evertsen & Johnsen, 2009). Apart from avoiding predators, however, the presence of kleptoplasts could provide additional advantages such as the translocating of photosynthates to kleptoplast-free animal cells. Recent studies, for example, demonstrated that carbon derived from photosynthesis is translocated to the reproductive organs of two different sea slugs (i.e., *Elysia timida* and *Elysia viridis*) ( Cartaxana *et al.*, 2021; Cruz *et al.*, 2020). The availability of “autotrophic powered cells” has multiple advantages for the animals as active photosynthesis can help in minimizing the loss of weight during food shortage, by supporting the reproductive output of the sea slugs or by increasing mucus production ( Cartaxana *et al.*, 2021; Lopes *et al.*, 2022; Maeda *et al.*, 2021; Shiroyama *et al.*, 2020; Yamamoto *et al.*, 2013).

Acquiring chloroplasts, however, may pose some problems to the animal cells: photosynthesis, especially under stronger light, is often associated with the production of reactive oxygen species (ROS). ROS mainly inhibit the *de novo* synthesis of proteins (i.e., impairing the repair of photosystem II (PSII)), but also damage DNA and non-photosynthetic host cells ( Murata *et al.*, 2007). To tackle damages, photosynthetic organisms are equipped with multiple photoprotective mechanisms that shield the photosynthetic apparatus from damages after light is absorbed by the light-harvesting systems. The prevalent mechanism is the conversion and dissipation of the excess excitation energy as heat, defined as the energy-dependent component of non-photochemical quenching (NPQ), qE. This fast reversible NPQ component depends on an irradiance-dependent proton gradient across the thylakoid membrane that decrease the lumenal pH. In land plants and algae, PsbS protein serves as the sensor that detects these changes and that activates qE quenching in PSII antenna by interacting with light harvesting complexes (LHCII) and inducing conformational changes ( Christa *et al.*, 2017; Dall’Osto *et al.*, 2005). qE is further modulated by the activation of a xanthophyll cycle (XC) (i.e., a process where violaxanthin (Vx) is reversibly converted to zeaxanthin (Zx) under high light through the intermediate conversion into antheraxanthin (Ax) by the enzymes violaxanthin de-epoxidase (VDE) and zeaxanthin epoxidase (ZEP) ( Goss & Jakob, 2010; Müller *et al.*, 2001). Apart from qE, authors hypothesized the presence of a pH-independent quenching mechanism, based on the direct binding of Zx to the periphery of antenna complexes. This NPQ component, named qZ, would appear later than qE and would use the Zx already synthesized and, as such, would be independent from lumen acidification and subsequent activation of VDE enzyme ( Dall’Osto *et al.*, 2005). A fast reversible NPQ component and functional XCs has been reported in kleptoplasts of *E. timida* and *E. chlorotica* ( Cartaxana *et al.*, 2019; Cruz *et al.*, 2015) as they are one of the main mechanisms present in the algal donors (i.e., *Acetabularia acetabulum* and *Vaucheria litorea*, respectively). Not all the macroalgae, however, have a functional XC and most of the protoprotective mechanism in this class of organisms are currently unclear. For example, reports suggest that some members of the order Bryopsidales lack a functional XC and do not show qE-type quenching during light stress, but instead accumulate other carotenoids (e.g., all- *trans*-neoxanthin) as a way to shield the reaction centers or to contribute to an efficient energy transfer between photosystem components ( Giossi *et al.*, 2020; Giossi *et al.*, 2021; Li *et al.*, 2023; Zuo *et al.*, 2006).

Conveniently, *E. viridis* is a sacoglossan sea slug that can eat a wide array of different macroalgae. In the Swedish coast, for example, it has been found foraging on multiple species such as the septate algae *Cladophora sericea*, *Cladophora rupestris*, *Chaetomorpha melagonium*, and *Ceramium virgatum*, as well as the siphonaceous species *Codium fragile* and *Bryopsis plumosa* ( Baumgartner & Toth, 2014). On the Portuguese coast, *E. viridis* can be found eating the alga *C. tomentosum* and this association, together with its photophysiology, has been extensively characterized ( Cruz *et al.*, 2014; Cruz *et al.*, 2015; Rauch *et al.*, 2018). *C. tomentosum* is one of the species that does not show a functional XC and, as such, *E. viridis* has never been reported to be able to functionally replicate such mechanism. Recently, in laboratory conditions, we could observe an active feeding behaviour of *E. viridis* on samples of *Chaetomorpha* sp. associated with the retention of functional chloroplasts. *Chaetomorpha* is structurally very different from *Codium* as it is a filamentous alga that can grow up to several centimeters in length. The thallus has a cylindrical shape, with multiple branches coming from the main filament. It is typically of a bright green colour but can sometimes appear darker or even blackish depending on the growth environment. Interestingly, the toughness of the outer cell wall seems also to be influenced by the intensity of the environmental light, assuming a rigid consistence when the alga is cultured under low irradiance ( Krause-Jensen *et al.*, 1996). Moreover, *Chaetomorpha* is reported to have a XC cycle (although its functionality has not been tested) and grown under high irradiances accumulates lutein, showing then a pigment composition different from *C. tomentosum* ( Bischof *et al.*, 2006; Christa *et al.*, 2017).

Taking advantage of these differences, in this work we compare the photophysiology of the sea slug *E. viridis* fed with the two different algae, investigating to which extent the xanthophyll cycle of the algal donor is conserved in the animals.

## Methods

### Animals and algae collection and maintenance

Wild sea slug *Elysia viridis* (Montagu, 1804) and the macroalga *Codium tomentosum* Stackhouse 1797, were collected during low tide in the intertidal rocky area of Aguda beach, Vila Nova de Gaia, Portugal (41°02′50.2″N, 8°39′15.2″W). A minimum of 100 animals were collected in several expeditions, depending on the tide and on the animal availability. The number was decided based on the dedicated space in the laboratory to ensure the best possible maintenance conditions. After collection, the animals and the algae were transported to the laboratory and maintained in 150 L recirculated life support systems (LSS) operated with artificial seawater (ASW) at 18 °C and a salinity of 35 ensured by a refrigerating system (Ultra Titan 200; HAILEA, GuangDong, China). A laboratory adaptation period of 2 weeks was chosen to ensure replicability in feeding and the light history of the animals at the beginning of the experiment. The photoperiod was set to 12 h light:12 h dark, with a photon scalar irradiance of 60–80 μmol photons m ^−2 ^s ^−1^, at the water surface, being provided by T5 fluorescent lamps. Irradiance was measured in the water with a US-SQS Spherical Micro Quantum Sensor and a ULM-500 Universal Light Meter (Walz, Effeltrich, Germany). *Chaetomorpha* sp. strands were obtained from coral life support systems existing in the same laboratory. Exact origin is unknown. The alga of this study was specifically cultivated in 2 L flasks with f/2 medium (without silica) and constant aeration at 20 °C and an irradiance of 60–80 μmol photons m ^−2^ s ^−1^ provided by LED lamps (Valoya 35 W, spectrum NS12), for 2 weeks before time zero of the experiment and up to the final feeding moment.

70 animals chosen to be used for the following evaluations were isolated in fish maternity nets in the same LSS with the above-described light conditions to ensure homogeneity of water conditions and a high degree of monitoring. The animals chosen among the original 100 sea slugs collected were the ones visually similar in size and color. Specimens were then provided with a constant supply of food (35 fed with either *C. tomentosum* or *Chaetomorpha* sp.) for 1 month. Approximately 2 gr of new algae were provided at least once every 5 days. Variables such as light distribution across the LSS and in different depths of the maternity net were considered providing that the two experimental settings were having the same characteristics. Every day, animals were monitored to ensure that no specimen was isolated in zones of the maternity net without algal food. The animals were weighted at the start and at the end of the feeding phase to estimate the daily weight increase by placing them on a nylon mesh with absorbent paper below, for gently removing the excess of water before weighting on an FH-200 laboratory scale (Waagenet AF GmbH, Berlin, Germany).

### Photosynthetic measurements

Fluorescence measurements were carried out on the samples by using an Imaging-PAM fluorometer (Mini version, Heinz Walz GmbH). To efficiently perform the measures on the sea slugs avoiding movements, the animals were blocked in a 0.2% agar solution in ASW before starting the measurements. The solution was prepared by mixing the appropriate concentration of agar in ASW, boiling the mixture, and letting it cool on ice. The solution was collected in an ice-cooled Pasteur pipette, slowly poured on the animals in a petri dish and solidified on a bed of ice before taking the measurements. At the end of the experiment, the animals were carefully removed from the embedding agar by using a Pasteur pipette and moved to a container with ASW to let them recover. They took approximately 15 min to recover full mobility. Effective quantum yield of photosystem II (ΦPSII) was measured as ΔF/Fm’, where ΔF corresponds to Fm’- F (the maximum minus the minimum fluorescence of light-exposed organisms). Maximum quantum yield of PSII, Fv/Fm, was calculated as (Fm-Fo)/Fm, where Fm and Fo are, respectively, the maximum and the minimum fluorescence of dark-adapted samples. For dark acclimation, samples were incubated for at least 30 min in darkness to allow the full relaxation of photosystems. Light curves were constructed with 12 incremental steps of actinic irradiance (E; 0, 15, 37, 55, 82, 136, 176, 238, 371, 446, 538, 649 µmol photons m ^-2 ^s ^-1^). For each step, ΦPSII was monitored every minute and electron transport rate (ETR) was calculated as E x ΦPSII x 0.84 x 0.5 (where 0.84 is the light absorptance by an average green leaf and 0.5 is the fraction of absorbed quanta available for PSII). The light response and associated parameters ETRm (maximum electron transport rate), Ek (light saturation coefficient) and alpha (photosynthetic rate in light limited region of the light curve) were characterized by fitting iteratively the model of the rETR versus E curves using MS Excel Solver ( Platt *et al.*, 1980). The fit was very good in all the cases (r>0.98). The NPQ kinetics were obtained by recording chlorophyll *a* fluorescence values in samples exposed to a light stress and recovery (LSR) protocol: sequential periods of 15 min dark, 20 min high light (HL, 1200 μmol photons m ^−2^ s ^−1^) and 40 min recovery under low light (LL _Rec_, 40 μmol photons m ^−2^ s ^−1^). NPQ was calculated as (Fm-Fm’)/Fm’ where Fm is the maximum fluorescence obtained in dark adapted samples while Fm’ is the maximum fluorescence recorded in any subsequential saturating light pulse. qE, qZ (qM) and qI components of NPQ were estimated as described in Sello *et al*. (2019) by fitting the relaxation kinetic with a biexponential decay (y=y0+A1 e ^−x/t1^+ A2 e ^−x/t2^) in the SigmaPlot software. Y0, the minimum value reached by the fitting, represented the NPQ that is not relaxed after 20 min of dark recovery and thus represents an estimation of the slow qI component. A1 and A2 instead corresponded to the fastest qE and qZ (or qM) components.

### Pigments analysis

Pigment analysis was performed as described by Cruz *et al*. (2014). Briefly, sea slugs and algae were sampled and immediately frozen in liquid nitrogen. Samples were freeze-dried, powdered by using a fine metal rod and pigments extracted in 95% cold buffered methanol (2% ammonium acetate) by adding the buffer, sonicating the samples for 2 min and then incubating them at -20°C for a minimum amount of 20 min. Sample debris were then removed by passing the samples through a 0.2 mm Fluoropore membrane filters (Millipore, Billerica, MA, USA). After filtration, the extracts were injected into a Prominence i –LC-2030C 3D Plus HPLC system (Shimadzu, Kyoto, Japan) equipped with the proprietary inbuilt photodiode array detector (PDA). Pigments were identified from absorbance spectra and retention times and concentrations were calculated in comparison with pure crystalline standards (DHI, Hørsolm, Denmark). The activity of the xanthophyll cycle and the sequential de-epoxidation of the pigments violaxanthin (Vx) to antheraxanthin (Ax) and zeaxanthin (Zx), was estimated by calculating the de-epoxidation state (DES) as: DES=([Zx]+0.5×[Ax])/([Zx]+[Ax]+[Vx]).

### Softwares and statistical analysis

Raw data obtained from the ImagingWin (WALZ, chlorophyll *a* variable fluorescence) and from Shimadzu LabSolution softwares were exported and analysed in Microsoft Excel (Microsoft Corporation, 2018. Available at: https://office.microsoft.com/excel). The same software has been used to build the figures and to calculate statistically significant differences by using independent samples t-tests.

## Results and discussion

Data underlying the results can be found on BioStudies ( Morelli, 2023).

### *Elysia viridis* mirrors the photosynthetic performance and the pigment profile of the different algal donors

*Elysia viridis* is a polyphagous species (i.e., it can feed on multiple algae) contrary to other members of the same genus such as *E. timida* or *E. chlorotica* that can feed and retain chloroplasts only from one specific algal food ( Baumgartner & Toth, 2014). Among the wide range of algal food, the slug is predominantly found on *C. tomentosum* or *C. fragile*, two coenocytic algae whose cells lack septation facilitating ingestion of cytoplasm by the animals. Sea slugs collected in the wild on the Portuguese coast are typically associated to *C. tomentosum*, thus, to carry out the experiments, we maintained them either on this alga or we provided them with abundant *Chaetomorpha* to induce a diet switch. In sacoglossans, the radula features a single row of teeth that is adapted to the group's suctorial feeding habits. Since the algal food exhibit a wide range of characteristics, such as toxic substances or heavy calcification, polyphagous species, such as *E. viridis*, have a high level of plasticity in form and dimension of teeth depending on the food source ( Jensen, 1993). When an animal is transferred from one alga to another, there is a learning span of at least one week, during which the morphology of the radular teeth is not distinguishable from that present with the previous food source ( Jensen, 1989; Rauch *et al.*, 2018). This learning process involves increased efficiency in grasping the food, piercing the algal cell, and sucking out the sap. Accordingly, when the animals were transferred from their natural habitat (where they mostly feed on *C. tomentosum*) to *Chaetomorpha*, the animals spent between 3 to 5 days only crawling on the alga filaments. The sea slugs became then more static between 7 and 10 days after being transferred, likely coinciding with the change of teeth and the process of learning how to pierce the cell wall, and feed properly. This plasticity corroborates previous observations ( Jensen, 1989), where specimens of *E. viridis* found on *Chaetomorpha* and moved on to *Codium* were able to efficiently learn how to feed on the latter alga. The animals, however, required a relatively longer time to learn again how to properly handle *Chaetomorpha* once transferred back to the original food source ( Jensen, 1989).

**Figure 1.**
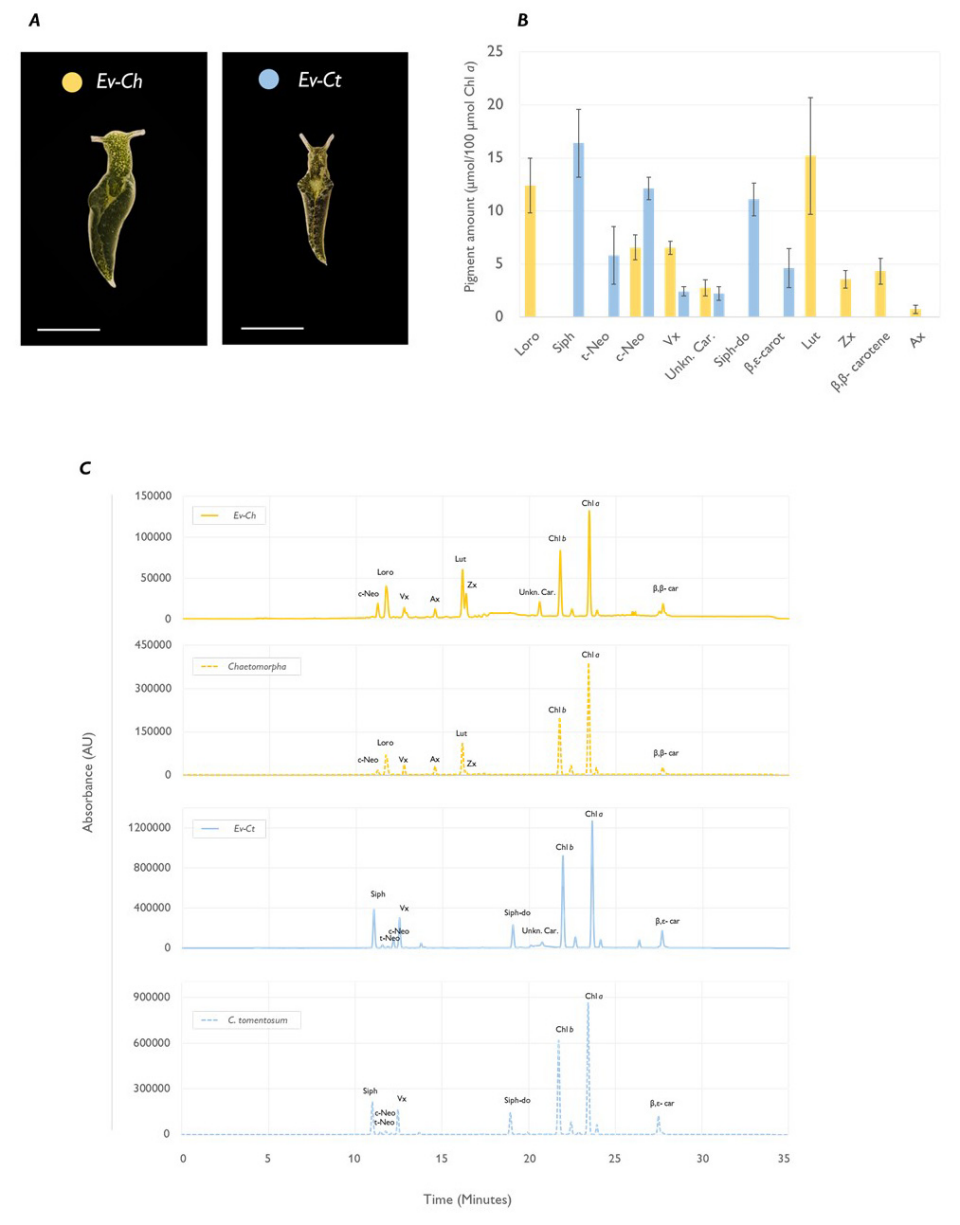
Pigment profile of *Elysia viridis* and its algae food sources. ( **A**) Representative pictures of *E. viridis* specimens fed with *Chaetomorpha* sp. (Ev-Ch) or with *C. tomentosum* (Ev-Ct). The scale bar corresponds to 0.5 cm; ( **B**) Carotenoid amount in sea slugs fed with the different algae. Data corresponds to mean and standard deviation (n=4) and values are expressed as relative to 100 µmol of Chl *a* in the same samples; ( **C**) HPLC chromatograms extracted at 440 nm, representative of sea slugs (straight line) and macroalgae (dotted line) pigment profiles. Siph, siphonaxanthin; *t*-Neo, all- *trans*-neoxanthin; *c*-Neo, 9′- *cis*-neoxanthin; Vx, violaxanthin; Ax, antheraxanthin; Zx, Zeaxanthin; Lut, Lutein; Siph-do, siphonaxanthin dodecenoate; Chl *b*, chlorophyll *b*; Chl *a*, chlorophyll *a*; β,ε-Car, β,ε-carotene; β,β-car, β,β-carotene; Unkn. Car., unidentified *Elysia viridis* carotenoid.

After one month of continuous feeding, the specimens fed on *Chaetomorpha* (Ev-Ch) showed a clearly different colour compared to the ones fed on *C. tomentosum* (Ev-Ct) ( Figure 1A). Chromatographic analysis associated the different shades of green with an altered pigment profile: Ev-Ct specimens showed high levels of siphonoxanthin and β,ε-carotene, typical of Bryopsidales algae ( Giossi *et al.*, 2021), while specimens fed with *Chaetomorpha* had no trace of the abovementioned carotenoids and accumulated instead high levels of lutein, β,β-carotene and loroxanthin ( Figure 1B). Moreover, we foundon both Ev-Ct and Ev-Ch an unknown carotenoid in animal samples already reported in other studies ( Cruz *et al.*, 2014) with the maximum absorption of 460 nm, with a retention time of about 23 min ( Figure 1C).

While the nature of this pigment is currently unclear, it could be a compound synthesized by the animal and necessary for its own metabolism that needs a common carotenoid precursor present in *C. tomentosum* and in *Chaetomorpha*. The carotenoid biosynthetic pathway is well characterized in plants and algae, occurring mainly in the chloroplasts, but it is absent in the animals that must obtain these compounds through their diets ( Morelli & Rodriguez-Concepcion, 2023). Indeed, the use of algae-derived carotenoids as precursors for keto-carotenoids necessary for animal metabolism is not so uncommon in molluscs. Sea slugs such as *Clione limacina* and *Paedoclione doliiformis*, oxidatively metabolize dietary carotenoids and accumulate them in their gonads to protect against oxidative stress ( Borodina & Zadorozhny, 2021; Maoka *et al.*, 2014), while specimens of *E. timida* were hypothesized to accumulate carotenoid photoreactive pigments in the eyespots used for light perception ( Rahat & Monselise, 1979).

**Figure 2.**
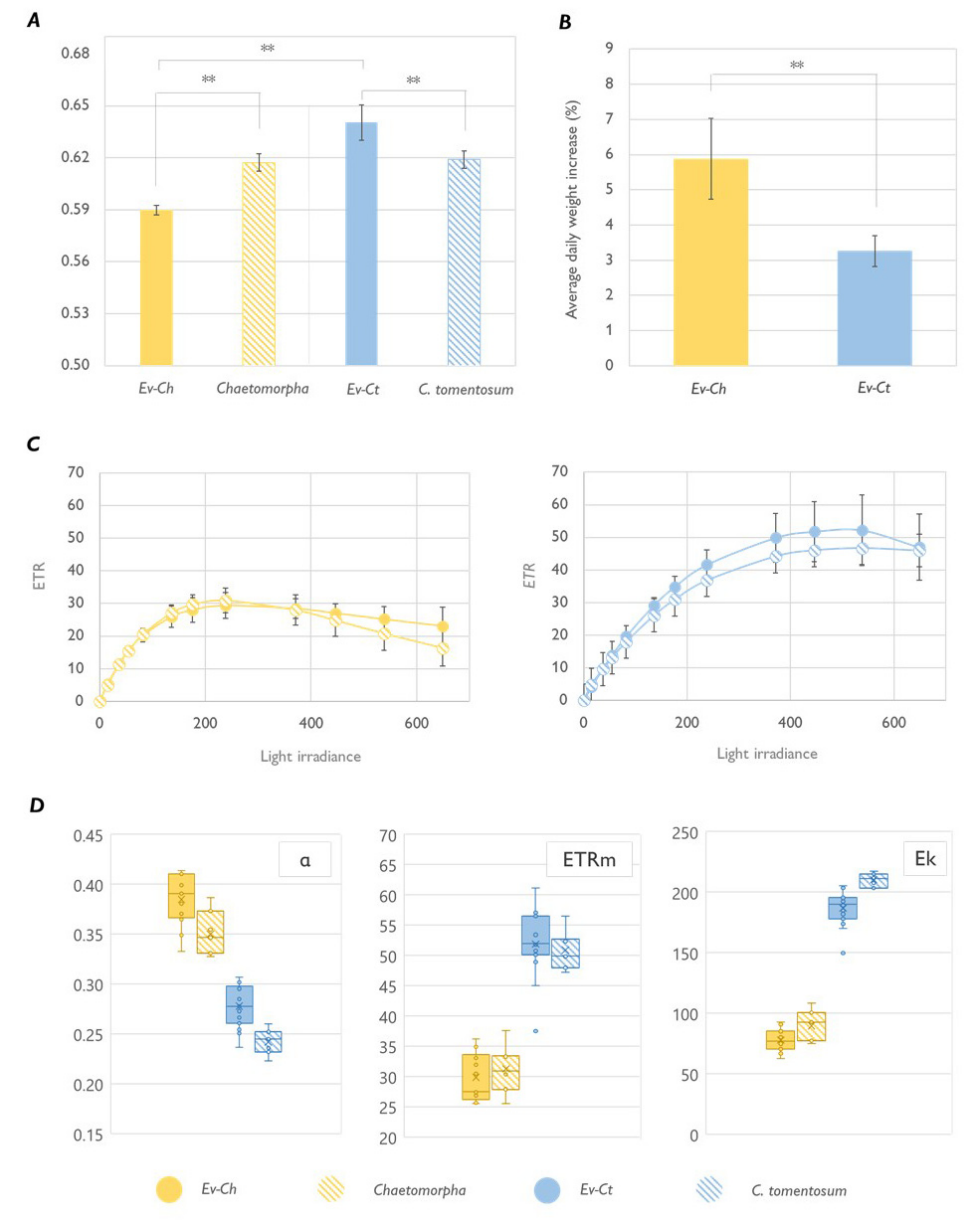
Photosynthetic performance of *Elysia viridis* and its algae food sources. ( **A**) Maximum quantum yield (Fv/Fm) in *E. viridis* fed with *Chaetomorpha* sp. (Ev-Ch), *Chaetomorpha* sp., *E. viridis* fed with *Codium tomentosum* (Ev-Ct) and *C. tomentosum*. Data correspond to mean and standard deviation (n=5); ( **B**) Averaged daily weight increase estimated for feeding animals, data corresponds to mean and standard deviation (n=10) measured after 1 month of continuous feeding; ( **C**) Light curves of sea slugs and the respective algal food. Data corresponds to mean and standard deviation (n=10); ( **D**) alpha, ETRm and Ek parameters calculated from the LCs represented in panel **C**. Boxes show the values between the upper and the lower quartile, the cross represents the mean and the horizontal line the median. Whiskers (the upper and lower extremes) and circles represent single data and the ones located outside of the whiskers limit are the outliers (data with the same numerical value are visualized as a single point). Asterisks mark statistically significant differences (t-test, ** p < 0.01).

Apart from the pigmentation, sea slugs photosynthetic performance was differently influenced by the algal food. The maximum quantum yield (Fv/Fm) of Ev-Ch specimens was significantly lower compared to the one observed in Ev-Ct animals. Interestingly, while the Fv/Fm in Ev-Ct slugs was higher than the one of *C. tomentosum* itself, Ev-Ch samples showed a lower value compared to the one of *Chaetomorpha* sp. ( Figure 2A). As already reported in other works, photosynthetic performance of sea slugs tends to be better than that of algae they are fed on, likely because of an intracellular supply of respired inorganic carbon and nutrients produced by the metabolism of the host ( Serôdio *et al.*, 2014). In the case of *Chaetomorpha* chloroplasts, they could be functional but losing stability very fast, hindering their performance once integrated in the host cells. The lifespan of *Chaetomorpha-*derived kleptoplasts in *E. viridis* tissues is unknown. However, a link between the different algal food and the retention performance in this species has already been described, with sea slugs being photosynthetically competent once fed with *Bryopsis hypnoides* but unable to incorporate chloroplasts from *Cladophora* sp. ( Rauch *et al.*, 2018). When fed with the former, or with *C. tomentosum*, in fact, *E. viridis* is classified as a long-term retention (LtR) species while it does not show traces of photosynthetic activity while fed with *Cladophora* sp. ( Rauch *et al.*, 2018). We can hypothesize that the incorporation process has a critical impact on the stability of the kleptoplasts. This is particularly evident if we consider species that can integrate only non-functional plastids such as *P. dendritica*. In the latter, plastids are never seen within depressions or pits in the cell surface prior to or during engulfment, but instead are phagocytized by cytoplasmic structures produced outward from the cell surface ( Evertsen & Johnsen, 2009; Mclean, 1976). The phagocytic activity observed in *P. dendritica* resembles somewhat the formation of food cups in the amoeba *Chaos chaos* and sometimes leave behind traces of intact chloroplasts that can provide a weak stable photosynthetic signal but just for few hours and up to 3 days ( Klochkova *et al.*, 2010).

To classify the photosynthetic state of the kleptoplasts, we calculated light curves (LCs) in *E. viridis* specimens and in their respective algal foods ( Figure 2C). Photosynthetic parameters estimated by plotting the data according to the model of Platt ( Platt *et al.*, 1980) showed a significantly higher maximum electron transport rate (ETRm) and light saturation coefficient (Ek) for Ev-Ct individuals and *C. tomentosum*, while the photosynthetic rate in the light-limited region of the light curve (alpha) was higher in Ev-Ch sea slugs and *Chaetomorpha* ( Figure 2D). More importantly, the LCs obtained from the animals were similar to the ones obtained from the algal food, confirming that chloroplasts are functional in the animal cells and that their photoacclimation state is inherited by the algae and maintained in the animal, as observed in other similar studies ( Cartaxana *et al.*, 2018). Curiously, Ev-Ch sea slugs shown a higher daily weight increase compared to Ev-Ct ones ( Figure 2B). We could speculate that sea slugs continuously feeding on *Chaetomorpha* could retain chloroplast for less time in their body (likely because of the poor photosynthetic performance) and would eat more algae when provided with unlimited food. In fact, it has been observed that in animals fed continuously the kleptoplast turnover tends to accelerate, reaching the exchange of 75% of the total plastids in a span of 9 days, while during starvation only 15% of total plastids are lost and digested ( Evertsen & Johnsen, 2009). While there are no studies comparing the nutrient content of the two algae, we could hypothesize a correlation between the weight increase and a faster direct degradation of kleptoplasts in Ev-Ch compared to Ev-Ct. The former sea slugs could adopt a binge eating habit to have a constant caloric intake instead of focusing on the integration of intact plastids in their body similarly to what happens in juveniles of *E. timida* that before establishing kleptoplasty feed continuously and digest chloroplasts to support their own body growth ( Laetz & Wägele, 2017).

### *Elysia viridis* fed with *Codium tomentosum* can maintain chloroplast photoprotective mechanisms, while animals fed with *Chaetomorpha* sp. cannot 

One of the main unresolved questions when investigating kleptoplasty is how plastids can continue to function in animal cells for long periods even if they are exposed constantly to wide range of stress conditions and without the access to many fundamental algal nuclear-encoded proteins. In particular, the conservation of mechanisms to tackle light stress in these photosynthetic animals is still puzzling ( Cruz & Cartaxana, 2022). Sacoglossan sea slugs are known to contribute to chloroplast shielding, either by folding their parapodia under high irradiances (i.e., to shield internal kleptoplasts while permitting filtered light energy to reach kleptoplasts on the parapodial undersides) or by simply crawling away from excessive light ( Cartaxana *et al.*, 2018; Donà *et al.*, 2022). Furthermore, physiological mechanisms involved in chloroplast protection can be maintained when the plastids are transferred to the animal cells. Some of the algae currently used as a food by sacoglossan sea slugs, indeed, are very resilient and show robust chloroplasts able to survive in harsh conditions. For example, isolated thylakoids of *V. litorea* were found to be more resilient to photoinhibition of PSII than spinach thylakoids, likely because of a lower ^1^O _2_ production and higher rates of ROS detoxification (e.g., through the action of α-tocopherol and carotenoids) ( Havurinne *et al.*, 2022). In some algae, the active conversion of Vx to Zx under high irradiances (i.e., the xanthophyll cycle) has been reported as one of the main mechanisms to protect against light stress and in some animals fed with these algae (e.g., *E. timida* and *E. chlorotica*) the cycle is maintained during medium periods of starvation and actively participate in kleptoplast protection ( Cartaxana *et al.*, 2019). However, other sea slugs (i.e., *E. cornigera*), fed with the same *A. acetabulum* were reported to show very different degree of kleptoplast compatibility being unable to cope with ROS accumulation with the same efficiency as *E. timida*( de Vries *et al.*, 2015). We hypothesized, thus, that the efficacy of the photoprotective mechanisms is not depending only on the physiologic characteristics of the algal donor but also on the individual compatibility of the stolen plastids with the sea slugs species.

**Figure 3.**
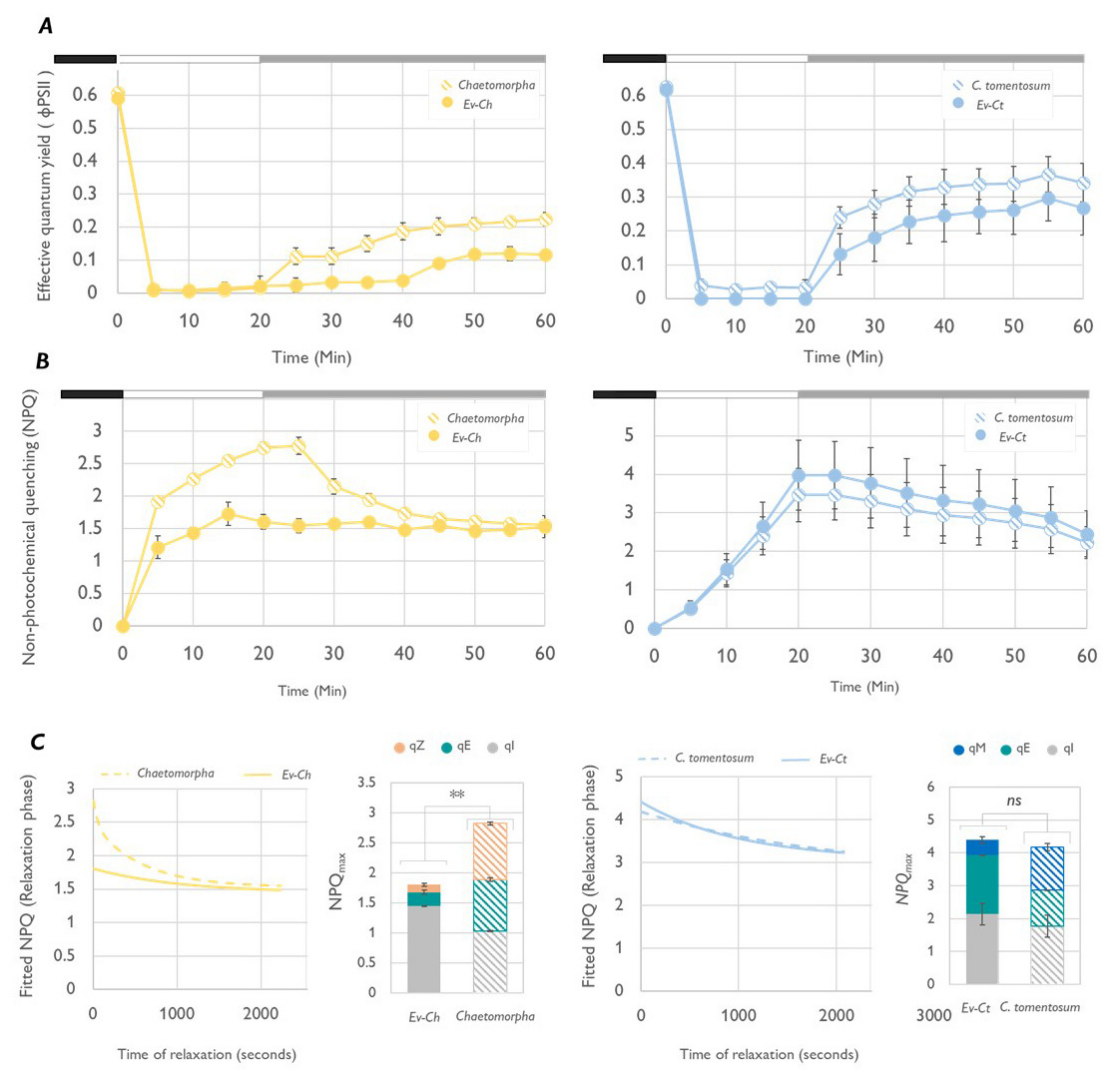
Light stress and recovery of *Elysia viridis* and its algae food sources. ( **A**) Effective quantum yield (ΦPSII) calculated in *E. viridis* fed with *Chaetomorpha* sp. (Ev-Ch), *Chaetomorpha* sp., *E. viridis* fed with *Codium tomentosum* (Ev-Ct) and *C. tomentosum* during a light stress-recovery experiment. In the chart, the different phases of the protocol are identified by the black box (15 min, previous dark acclimation), the white box (light stress, exposure to 1200 μmol photons m ^-2^ s ^-1^ for 20 min) and the grey box (low light recovery, 40 μmol photons m ^-2^ s ^-1 ^for 40 min). Data corresponds to mean and standard deviation (n=5); ( **B**) Non-photochemical quenching (NPQ) kinetics obtained in the same conditions as described for panel A; ( **C**) Graphs representing the relaxation phase of the NPQ kinetic fitted to the equation of a bi-exponential decay in SigmaPlot. The program is used also to calculate the components of the NPQ represented in the two bar charts as part of the total NPQ level. Asterisks mark statistically significant differences (t-test, ** p < 0.01, ns: no significant differences) between the values of total NPQ _max_ in the indicated samples.

**Figure 4.**
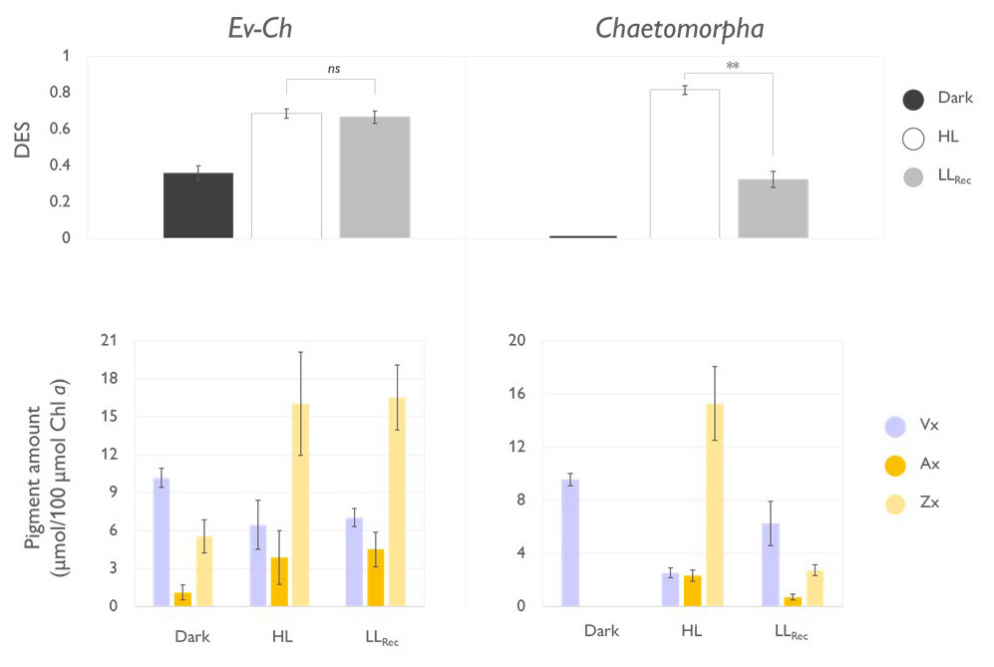
Operation of the xanthophyll cycle in *Elysia viridis* fed with *Chaetopmorpha* sp. (Ev-Ch) and in the individual algae. The top panels show the de-epoxidation state (DES) expressed as (Zx+0.5*Ax)/(Vx+Ax+Zx) in Ev-Ch sea slugs and *Chaetomorpha* sp. subjected to the light stress and recovery protocol represented in Figure 3; Vx, violaxanthin; Ax, antheraxanthin; Zx, Zeaxanthin. The lower panel represent the levels of the single xanthophylls expressed as µmol of pigment relative to 100 µmol of Chl *a* observed in the same samples. Data corresponds to mean and standard deviation (n=4). Asterisks mark statistically significant differences between the high light (HL) and the recovery in low light (LL _Rec_) phases (t-test, ** p < 0.01, ns: no significant differences).

To test our hypothesis, we evaluated the response to short term light stress in Ev-Ct and Ev-Ch specimens and in the respective food sources. Under high light (HL, 1200 μmol photons m ^-2^ s ^-1^), the photosynthetic yield ΦPSII of the sea slugs reached values very near 0, showing an expected photoinhibition state, regardless of the algal food ( Figure 3A). At the same time NPQ level increased to be then slowly dissipated under low irradiances (LL _Rec_, 40 μmol photons m ^-2^ s ^-1^) in concomitance with a recovery of photosynthetic yield ( Figures 3A, B). The relaxation and recovery kinetics followed the same pattern in sea slugs and algae and, as observed in other studies, 40 min of LL incubation were not enough to recover the maximum quantum yield ( Cruz *et al.*, 2015). The slow relaxation of the NPQ in *C. tomentosum* could be influenced by chloroplast movement inside the algal tissue as chloroplasts can redistribute and reorientate in the cell as a response to the light direction resulting in typical patterns for light of low or high fluence rate. In this condition, plastids take a “high intensity arrangement” designed to protect the chloroplasts against excessive radiation by stacking the plastids on the cell borders and piling them to provide self-shading ( Davis & Hangarter, 2012). In *C. tomentosum*, which has no functional XC, chloroplast movement can have an important role on preventing excess energy absorption. Moreover, the kinetic of NPQ relaxation under low light in *C. tomentosum* and Ev-Ct is strikingly like the one observed in *A. thaliananpq4* mutants. The latter lack the PsbS protein and are unable to carry on a functional xanthophyll cycle thus rely on plastid movement to develop a slow, qE-type, energy quenching mechanism named qM ( Dall’Osto *et al.*, 2014). Accordingly, we could mathematically calculate a high contribution of qM component to the quenching by fitting the relaxation kinetic to the equation of a biexponential decay ( Figure 3C). In fact, during NPQ relaxation, a pivotal role is played by the fast component qE that act in the first minutes of low light/dark exposure, but a slower component, associated with Zx binding to LHC proteins named qZ is the responsible for energy quenching after 10–15 min of relaxation ( Dall’Osto *et al.*, 2005). In organisms where this pigment is not present, such as *C. tomentosum* or the *npq4 A. thaliana* mutant, the dissipation of energy is related to chloroplast movement, hence the qM definition ( Dall’Osto *et al.*, 2012). On another hand, differently from *Codium*, *Chaetomorpha* accumulates Zx under high irradiances and its NPQ kinetic is very similar to the ones of other XC-performing algae such as *V. litorea* ( Cruz *et al.*, 2015). Despite this, as observed for *Codium*, also in *Chaetomorpha* the recovery of ΦPSII is not complete after 40 min in low light. However, the fast qE-quenching component in the first minutes is evident and, considering the presence of Zx, the subsequent slower recovery could be related to the qZ component. Interestingly, Ev-Ch animals showed the ability to build up NPQ up to a certain extent but were unable to relax it and show little recovery of the photosynthetic activity during LL _Rec_ ( Figures 4A, B).

When investigating the functionality of the XC, we observed that dark-acclimated *Chaetomorpha* sp. showed no traces of Zx, accumulated it after 20 min of HL exposure, and about 75% of Zx was converted back to Vx at the end of the LL _Rec _ ( Figure 4). The cycle resulted in a significantly higher de-epoxidation state (DES) during the HL exposure compared to the LL _Rec_. On the other hand, Ev-Ch sea slugs showed traces of Zx already upon dark acclimation, under HL the amount of this pigment increased, but did not decrease under LL _Rec_, accordingly with the inability to quench the built up of NPQ.

As previously observed, *Chaetomorpha* chloroplasts are less photosynthetically competent when integrated in *E. viridis*. For this reason, an excessive light exposure could result in severe photoinhibition. In this state, the proton gradient across the thylakoidal membranes could be altered and the cycle would not recover back to violaxanthin as the responsible enzymes would not find a suitable environment to function ( Tjus & Andersson, 1993). In the algae, this situation can be overcome by plastid-targeted proteins codified by nuclear encoded genes that likely *E. viridis* lacks. The possibility for horizontal gene transfer (HGT) from algal donor to the animal genome has been a highly debated possibility over the years. Earlier evidence of HGT from the alga *V. litorea* to the nuclear DNA of the sea slug *E. chlorotica*, in fact, was contradicted by reports of the absence of algal-derived genes in the germline of the sea slug while genomic and transcriptomic studies on the sea slugs *E. timida* and *P. ocellatus* found no evidence of HGT ( Bhattacharya *et al.*, 2013; Pierce *et al.*, 2012; Wägele *et al.*, 2011). On the other hand, experimental evidence in other kleptoplastidic organisms revealed that kleptoplasty is supported by nucleus-encoded genes for plastid-targeted proteins with specific targeting signals to the kleptoplasts and by the presence of plastid transporters to facilitate targeting of the proteins and metabolites into the kleptoplasts ( Hehenberger *et al.*, 2019; Lekieffre *et al.*, 2018). In some kleptoplastidic organisms, such as Ross Sea dinoflagellates (RSD) photosynthetic functions split between an ancestral plastid (i.e., peridinin plastid) and the kleptoplasts. In these organisms the electron flow from PSII to PSI is lost during plastid sorting and is recovered through some proteins codified in the dinoflagellate nucleus and then targeted to the kleptoplast through a specific signal ( Hehenberger *et al.*, 2019). We hypothesized that when *E. viridis* feeds on an alga that is not its “preferred” donor (e.g., *Chaetomorpha*), the compatibility would be only partial and even if the kleptoplasts can maintain certain functionalities in the animal cells (e.g., a basal photosynthetic activity), they would be unable to cope with stress as the codification and import of specific proteins would be required. Transit peptide diversity across plant kingdom is very significant and proteins codified in a plant species sometimes cannot enter a chloroplast of a different organism without minor changes in the coding sequence ( Patron & Waller, 2007). A kleptoplast-targeted protein codified in *E. viridis* but programmed to enter *Codium* chloroplasts, thus, could be unable to interact with *Chaetomorpha* plastids and to resolve fundamental functions in this chloroplast such as its repair or metabolism regulation, leading to its fast decay. Moreover, the mechanism for kleptoplast integration in animal cells is a debated question, as in some organisms they are found surrounded by an additional membrane likely remaining from the phagosome, while in other studies this additional membrane is absent ( Martin *et al.*, 2013). Modifications of the inner and outer envelope during the incorporation of the kleptoplasts in the animal cells could affect the capacity of specific protein to enter the plastid. The association *E. viridis – Codium* likely was specifically established over the evolution and results in the codification of all the necessary subunits of a functional translocon complex capable of importing useful proteins to maintain the functionality of the chloroplast. In the case of *Chaetomorpha* chloroplasts, some of these components could be missing or could interact differently with the slugs nuclear-encoded proteins resulting in a loss of function.

## Conclusions

This study assesses the photophysiology of the sea slug *E. viridis* fed with algae with different photoprotective and photosynthetic capabilities ( *C. tomentosum* and *Chaetomorpha* sp.). In this work, we took advantage of the physiological differences of the chloroplast algal donor to investigate what mechanisms are conserved in the animals. Our results show that specific photoprotective mechanisms of algal chloroplasts (e.g., the xanthophyll cycle) are not necessarily maintained once the plastids are transferred to the sea slug cells. This work questions the actual interaction between the photosynthetic sea slugs and the integrated kleptoplasts and suggest that plastid functionality depends on the sea slug-alga association. These results link the phenomenon of kleptoplasty in *Elysia* to the presence of a protein targeting system established during the early stage of endosymbiosis that likely helps in supporting transient organelles such as the kleptoplasts ( Hehenberger *et al.*, 2019). Algae chloroplasts have their individual robustness but, once part of the host animal cell, require a support that only some can provide. Thus, only in case of specific associations, such as the ones between *E. timida* and *A. acetabulum* or *E. chlorotica* and *V. litorea*, it would be possible for the kleptoplasts to maintain all its functionalities, likely because of a coevolution that allowed the specific interaction between the animal physiology and the one of a particular type of plastid. 

## Ethics and consent

The use of *E. viridis* specimens was not subjected to ethical approval considering that the use of lower level invertebrates species (e.g., brine shrimp, fruit flies, nematodes, mosquitoes, etc.) is not restricted for research purposes. After the collection, animals were maintained in optimal conditions using the LSS in controlled environment to ensure the maximum fitness. Animals were subjected to starvation only when required by the experimental setting.

## Data Availability

BioStudies: Datasets accompanying the manuscript: "Food shaped photosynthesis: Photophysiology of the sea slug Elysia viridis fed with two alternative chloroplast donors". Accession number S-BSST1106, https://www.ebi.ac.uk/biostudies/studies/S-BSST1106 ( Morelli, 2023).
