# Supplementary material for: Food shaped photosynthesis: Photophysiology of the sea slug Elysia viridis fed with two alternative chloroplast donors
Source: Open Res Eur. 2024 Mar 13;3:107. Originally published 2023 Jun 28. [Version 2] doi: 10.12688/openreseurope.16162.2 (PMC11079582; doi:10.12688/openreseurope.16162.2)
Supplement: Peer review history

# Reviewer report: Reviewer response for version 2

**DOI:** 10.21956/openreseurope.17955.r38910

**Published:** 2024-05-08

**Authors:** Rachael M Wade

**Affiliations:** 1 The University of British Columbia, Vancouver, British Columbia, Canada

I believe the authors have responded well to the revisions and made them satisfactorily.

Is the study design appropriate and does the work have academic merit?

Yes

Is the work clearly and accurately presented and does it cite the current literature?

Partly

If applicable, is the statistical analysis and its interpretation appropriate?

Yes

Are all the source data underlying the results available to ensure full reproducibility?

Yes

Are the conclusions drawn adequately supported by the results?

Partly

Are sufficient details of methods and analysis provided to allow replication by others?

Yes

Reviewer Expertise:

Algal evolution and biology; plant physiology; sacoglossan kleptoplasty

I confirm that I have read this submission and believe that I have an appropriate level of expertise to confirm that it is of an acceptable scientific standard.

# Reviewer report: Reviewer response for version 2

**DOI:** 10.21956/openreseurope.17955.r38911

**Published:** 2024-04-12

**Authors:** Yoichi Yusa

**Affiliations:** 1 Faculty of Science, Nara Women's University, Nara, Nara Prefecture, Japan

I have no further comments.

Is the study design appropriate and does the work have academic merit?

Yes

Is the work clearly and accurately presented and does it cite the current literature?

Yes

If applicable, is the statistical analysis and its interpretation appropriate?

Yes

Are all the source data underlying the results available to ensure full reproducibility?

Yes

Are the conclusions drawn adequately supported by the results?

Yes

Are sufficient details of methods and analysis provided to allow replication by others?

Yes

Reviewer Expertise:

Aquatic ecology

I confirm that I have read this submission and believe that I have an appropriate level of expertise to confirm that it is of an acceptable scientific standard.

# Reviewer report: Reviewer response for version 1

**DOI:** 10.21956/openreseurope.17448.r33598

**Published:** 2023-07-25

**Authors:** Rachael M Wade

**Affiliations:** 1 The University of British Columbia, Vancouver, British Columbia, Canada

**General comments:**

 The authors consistently cite their own papers, when there are often a multitude of other papers by other authors that could be cited. I would appreciate seeing the paper revised through this lens. For example, recent work by the authors is cited when discussing the proposed lack of the xanthophyll cycle in some siphonous greens, but this was supported by Benson and Cobb (1983) ^1^. There are also consistent issues with incorrectly crediting and citing papers, please carefully review each paper referenced and its findings.

**Introductory summary sections:**

*Background:*

- I think the first sentence could be reworded to be more clear – Some sacoglossan sea slugs integrate chloroplasts from the algae they feed on into their cells (perhaps add here "where they continue to function photosynthetically"), a process termed kleptoplasty. Chloroplasts are inherently intracellular, so the current wording does not truly describe the process of kleptoplasty.
- 'Associated with' instead of 'associated to'.

*Results:*

- Add 'similarly' or 'comparably' after "While sea slugs fed with *C. tomentosum* react…"
- Change 'algae' to 'alga'.

*Conclusions:*

- This conclusion is too strong given the methods used in this study; no attempts to understand the role of the slug as a new host plays in interacting with the different chloroplasts, only how the chloroplasts are functioning in the host. There are significant evolutionary and biological differences between *Codium* and *Chaetomorpha*, they are from two completely different Orders. This taxonomic difference in host species is reflected in the genomes of the algae – the cladophoralean genome is a fragmented assemblage of hairpin chromosomes (see Del Cortona *et al.* 2017 *Cell* ^2^). This may be playing a significant role in how the chloroplasts behave independent of their algal cells. Additionally, siphonous greens have siphonein and siphonoxanthin, which may play a role in photoprotection (although they’re largely described as antennae accessory pigments). Your conclusion is entirely plausible, but the possibility of evolutionary history and biology is also equally plausible and need to be discussed as such. This is abundantly clear when so much of this theory is based on reorganization of chloroplasts as a strategy to reduce photodamage, but this strategy is common in higher land plants, it is not clear to me why the authors assume that *Chaetomorpha* would not be able to exhibit similar phototactic responses. I do feel that the Conclusion section does a better job of supporting some of the theories I question here than other parts of the manuscript and could be used to strengthen those sections in question.

**Introduction:**

*First paragraph:*

- Change 'associated' to 'attributed'.
- Your citing of your own 2022 paper for the definition of kleptoplasty and its occurrence in Sacoglossa is not correct, you should be citing the papers by those first witnessed the phenomenon in sacoglossans, which is most often attributed to Kawaguti and Yamasu (1965) ^3^ and Greene (1970) ^4^, Green & Muscatin (1972) ^5^. If you want to say the topic is reviewed by your paper, great, but give credit where credit is due. Particularly because, surprisingly, none of those papers above are cited in that review.
- Your description of the extent of the digestive tissue and the crypsis it results in only applies to some taxa. Please either revise to be more inclusive of the diversity of Sacoglossa morphologies, or narrow your discussion entirely to your study organism.
- I have never heard this theory before, that kleptoplasty arose to achieve crypsis. To me, this does not make sense in reflection of endosymbiotic theory, in which most people describe kleptoplasty as a sort of steppingstone to true endosymbiosis. I’ve re-read the Evertsen & Johnsen 2009 publication you cited and nowhere does this paper test this theory, nor even suggest it. The next sentence is even more confusing when you say that a sacoglossan with non-functional kleptoplasts is still benefiting from photosynthate! How? This is perhaps a transition issue, please revise for clarity.

*Second paragraph:*

- Donohoo *et al.* 2020 ^6^ and Evertsen & Johnsen 2009 ^7^ should also be cited in your last sentence.

*Fourth paragraph:*

- Would be helpful to note that *Ceramium* is a rhodophyte, while all other taxa mentioned are chlorophytes. Similarly, in the description of *Chaetomorpha*, it would be helpful to reiterate that while multinucleate, it is not siphonous, like *Codium.*

**Methods:**

*Second paragraph:*

- Change 'weighted' to 'weighed'.

**Results:**

- Defined polyphagous in its second appearance, rather than the first in the intro. Please revise.
- Change 'sap' to 'cytoplasm'.
- I’m confused by your discussion of *Placida*, I’m assuming in review of Evertsen & Johnsen (2009) ^7^ and describing them has having non-functional yet incorporated plastids. The abstract and results state that the chloroplasts are digested immediately, not maintained, yet non-functional. They are phagocytized first, but are immediately digested. The way their study is described in some (but not all) of this manuscript, it suggests the chloroplasts are somehow hanging out in the slugs tissues, alive, but not photosynthesizing. Additionally, this paper is credited with the observation that up to 75% of kleptoplasts are overturned, but this was actually studied by Gallop *et al.* 1980 ^8^.

*Paragraph 5:*

- Increased weight/decreased mortality at low-moderate light was also shown in Donohoo *et al. *2020 ^6^.

*Paragraph 6:*

- RE: siphonous greens exhibiting phototaxis in regard to plastid rearrangement: I don’t doubt it occurs, but I think it is just as likely to occur in a large-celled, septate green, like *Chaetomorpha*. This is also seen throughout higher land plants.

*Paragraph 7:*

- In my opinion, the HGT debate has been put to bed by the thorough work in Maeda *et al. *2021 ^9^ but is missing from your discussion of this topic.

Is the study design appropriate and does the work have academic merit?

Yes

Is the work clearly and accurately presented and does it cite the current literature?

Partly

If applicable, is the statistical analysis and its interpretation appropriate?

Yes

Are all the source data underlying the results available to ensure full reproducibility?

Yes

Are the conclusions drawn adequately supported by the results?

Partly

Are sufficient details of methods and analysis provided to allow replication by others?

Yes

Reviewer Expertise:

Algal evolution and biology; plant physiology; sacoglossan kleptoplasty

I confirm that I have read this submission and believe that I have an appropriate level of expertise to confirm that it is of an acceptable scientific standard, however I have significant reservations, as outlined above.

## References

[1] : Pigment/Protein Complexes of the Intertidal Alga Codium fragile (Suringar) Hariot. *New Phytologist* .1983;95(4) : 10.1111/j.1469-8137.1983.tb03523.x 581-594 10.1111/j.1469-8137.1983.tb03523.x

[2] : The Plastid Genome in Cladophorales Green Algae Is Encoded by Hairpin Chromosomes. *Curr Biol* .2017;27(24) : 10.1016/j.cub.2017.11.004 3771-3782.e6 10.1016/j.cub.2017.11.004 29199074

[3] : Electron microscopy on the symbiosis between an elysioid gastropod and chloroplasts of a green alga. *Biological Journal of Okayama University* .1965;11:57-65

[4] : Symbiosis in sacoglossan opisthobranchs: functional capacity of symbiotic chloroplasts. *Marine Biology* .1970;7(2) : 10.1007/BF00354917 138-142 10.1007/BF00354917

[5] : Symbiosis in sacoglossan opisthobranchs: photosynthetic products of animal-chloroplast associations. *Marine Biology* .1972;14(3) : 10.1007/BF00348288 253-259 10.1007/BF00348288

[6] : Finding the Sweet Spot: Sub-Ambient Light Increases Fitness and Kleptoplast Survival in the Sea Slug Plakobranchus cf. ianthobaptus Gould, 1852. *Biol Bull* .2020;238(3) : 10.1086/709371 154-166 10.1086/709371 32597715

[7] : In vivo and in vitro differences in chloroplast functionality in the two north Atlantic sacoglossans (Gastropoda, Opisthobranchia) Placida dendritica and Elysia viridis. *Marine Biology* .2009;156(5) : 10.1007/s00227-009-1128-y 847-859 10.1007/s00227-009-1128-y

[8] : The biology of chloroplast acquisition by Elysia viridis. *Royal Society* .1980;207(1169) : 10.1098/rspb.1980.0027 10.1098/rspb.1980.0027

[9] : Chloroplast acquisition without the gene transfer in kleptoplastic sea slugs, Plakobranchus ocellatus. *Elife* .2021;10: 10.7554/eLife.60176 10.7554/eLife.60176 33902812 PMC8079154

# Response

**Published:** 2023-07-30

**Authors:** Luca Morelli

Dear Dr. Wade, We really appreciate your comments and the constructive discussion that they arose. We will work on a version considering all your reviews and suggestions and we will upload it as soon as possible. 

 Luca Morelli

# Response

**Published:** 2024-02-17

**Authors:** Luca Morelli

**General comments:** **C1**: The authors consistently cite their own papers, when there are often a multitude of other papers by other authors that could be cited. I would appreciate seeing the paper revised through this lens. For example, recent work by the authors is cited when discussing the proposed lack of the xanthophyll cycle in some siphonous greens, but this was supported by Benson and Cobb (1983)1. There are also consistent issues with incorrectly crediting and citing papers, please carefully review each paper referenced and its findings. **R1**: We appreciate and thank the reviewer for all the comments and feedback. We have checked the chosen references to correct any inconsistencies and provided new references to credit other authors.   Introductory summary sections:  

**Background:** **C2**: I think the first sentence could be reworded to be more clear – Some sacoglossan sea slugs integrate chloroplasts from the algae they feed on into their cells (perhaps add here "where they continue to function photosynthetically"), a process termed kleptoplasty. Chloroplasts are inherently intracellular, so the current wording does not truly describe the process of kleptoplasty. 'Associated with' instead of 'associated to'.

**R2**: We have rephrased the sentence for improved clarity and done the requested corrections. It reads, “Some Sacoglossa sea slugs steal and integrate chloroplasts derived from the algae they feed on into their cells where they continue to function photosynthetically, a process termed kleptoplasty”  

**Results:** **C3:**

- Add 'similarly' or 'comparably' after "While sea slugs fed with C. tomentosum react…"
- Change 'algae' to 'alga'.

**R3**: We have done the requested corrections.  

**Conclusions:** **C4**: This conclusion is too strong given the methods used in this study; no attempts to understand the role of the slug as a new host plays in interacting with the different chloroplasts, only how the chloroplasts are functioning in the host. There are significant evolutionary and biological differences between Codium and Chaetomorpha, they are from two completely different Orders. This taxonomic difference in host species is reflected in the genomes of the algae – the cladophoralean genome is a fragmented assemblage of hairpin chromosomes (see Del Cortona et al. 2017 Cell2). This may be playing a significant role in how the chloroplasts behave independent of their algal cells. Additionally, siphonous greens have siphonein and siphonoxanthin, which may play a role in photoprotection (although they’re largely described as antennae accessory pigments). Your conclusion is entirely plausible, but the possibility of evolutionary history and biology is also equally plausible and need to be discussed as such. This is abundantly clear when so much of this theory is based on reorganization of chloroplasts as a strategy to reduce photodamage, but this strategy is common in higher land plants, it is not clear to me why the authors assume that Chaetomorpha would not be able to exhibit similar phototactic responses. I do feel that the Conclusion section does a better job of supporting some of the theories I question here than other parts of the manuscript and could be used to strengthen those sections in question.

**R4:** We have modified the Results and Discussion and Conclusion sections for clarity and to downgrade the conclusions of our study. In its current form, the manuscript focuses on the characterization of the photophysiology providing some ideas for future works. Any speculations are clearly identified as such.  

**Introduction:** First paragraph: **C5**: Change 'associated' to 'attributed'.

**R5**:  We have performed the required correction.

**C6**: Your citing of your own 2022 paper for the definition of kleptoplasty and its occurrence in Sacoglossa is not correct, you should be citing the papers by those first witnessed the phenomenon in sacoglossans, which is most often attributed to Kawaguti and Yamasu (1965)3 and Greene (1970)4, Green & Muscatin (1972)5. If you want to say the topic is reviewed by your paper, great, but give credit where credit is due. Particularly because, surprisingly, none of those papers above are cited in that review.

**R6**: We have inserted additional references in the text.  

**C7**: Your description of the extent of the digestive tissue and the crypsis it results in only applies to some taxa. Please either revise to be more inclusive of the diversity of Sacoglossa morphologies or narrow your discussion entirely to your study organism.

**R7**: we have modified the text to increase the clarity. Now it raeds, “While in some species of Sacoglossa sea slugs (e.g., *Elysia timida* and *Plakobranchus ocellatus*) kleptoplasts are mostly visible only in some body regions (e.g., the dorsal area), in other species such as E. viridis, they are distributed evenly and not concentrated in a specific part of the animal (Gallop et al., 1980). In E. viridis, the presence of ramifications of digestive tubules, throughout most of the sea slug’s body, contributes to a colour resembling that of its algal food and helps the animal to blend with the environment to escape predators (Hirose, 2005)”  

**C8**: I have never heard this theory before, that kleptoplasty arose to achieve crypsis. To me, this does not make sense in reflection of endosymbiotic theory, in which most people describe kleptoplasty as a sort of steppingstone to true endosymbiosis. I’ve re-read the Evertsen & Johnsen 2009 publication you cited and nowhere does this paper test this theory, nor even suggest it. The next sentence is even more confusing when you say that a sacoglossan with non-functional kleptoplasts is still benefiting from photosynthate! How? This is perhaps a transition issue, please revise for clarity.

**R8**: We have rephrased the section for clarity (see R7).   Second paragraph:

**C9**: Donohoo et al. 20206 and Evertsen & Johnsen 20097 should also be cited in your last sentence.

**R9**: The references suggested were added to the text. Fourth paragraph:

**C10**: Would be helpful to note that Ceramium is a rhodophyte, while all other taxa mentioned are chlorophytes. Similarly, in the description of Chaetomorpha, it would be helpful to reiterate that while multinucleate, it is not siphonous, like Codium.

**R10**: We have modified the text to include a short description of Chaetomorpha to offer a better overview of its morphology. Now it reads, “ *Chaetomorpha *is structurally very different from *Codium* as it is a filamentous alga that can grow up to several centimeters in length. The thallus has a cylindrical shape and is usually unbranched, loosely entangled and stiff, made by the junction of several squared cells separated by septa (Blair et al., 1982). This characteristic affects the feeding pattern of the animals that must move and pierce every single cell to suck the cytoplasm”.  

**Methods:** **C11**: Second paragraph: Change 'weighted' to 'weighed'.

**R11**: Corrected. Results:

**C12**: Defined polyphagous in its second appearance, rather than the first in the intro. Please revise.  Change 'sap' to 'cytoplasm'.

**R12**: We have made the required corrections.  

**C13**: I’m confused by your discussion of Placida, I’m assuming in review of Evertsen & Johnsen (2009) and describing them has having non-functional yet incorporated plastids. The abstract and results state that the chloroplasts are digested immediately, not maintained, yet non-functional. They are phagocytized first, but are immediately digested. The way their study is described in some (but not all) of this manuscript, it suggests the chloroplasts are somehow hanging out in the slugs tissues, alive, but not photosynthesizing. Additionally, this paper is credited with the observation that up to 75% of kleptoplasts are overturned, but this was actually studied by Gallop et al. 1980.

**R13**: The discussion of *Placida* was removed to avoid confusion as no actual data from experiments involving *Placida* were used for this work. Paragraph 5:

**C14:** Increased weight/decreased mortality at low-moderate light was also shown in Donohoo et al. 2020.

**R14:** The additional reference was inserted in the text Paragraph 6:

**C15:** siphonous greens exhibiting phototaxis in regard to plastid rearrangement: I don’t doubt it occurs, but I think it is just as likely to occur in a large-celled, septate green, like Chaetomorpha. This is also seen throughout higher land plants.

**R15: **We have rearranged the text to make our statements clearer. Now it reads, **“**In *C. tomentosum*, which has no functional XC, chloroplast movement could have a prevalent role on preventing excess energy absorption. In siphonous algae such as *Halimeda distorta*, the piling of chloroplasts to one side of the algae branch following prolonged exposure to very high irradiance or darkness is so intense to change the green color intensity (Drew & Abel, 1992). Moreover, the kinetic of NPQ relaxation under low light in *C. tomentosum* and Ev-Ct is strikingly like the one observed in *A. thaliana* npq4 mutants. The latter lack the PsbS protein and are unable to carry on a functional xanthophyll cycle thus rely on plastid movement to develop a slow, qE-type, energy quenching mechanism named qM (Dall’Osto et al., 2014). Accordingly, we could mathematically calculate a high contribution of qM component to the quenching by fitting the relaxation kinetic to the equation of a biexponential decay (Figure 3C). In fact, during NPQ relaxation, a pivotal role is played by the fast component qE that act in the first minutes of low light/dark exposure, but a slower component, associated with Zx binding to LHC proteins named qZ is the responsible for energy quenching after 10–15 min of relaxation (Dall’Osto et al., 2005). In organisms where this pigment is not present, such as *C. tomentosum* or the npq4 *A. thaliana* mutant, the dissipation of energy is related to chloroplast movement, hence the qM definition (Dall’Osto et al., 2012). On another hand, differently from *Codium*, *Chaetomorpha* accumulates Zx under high irradiances and its NPQ kinetic is very similar to the ones of other XC-performing algae such as *V. litorea* suggesting a more marginal role for chloroplast movement-based photoprotective mechanisms over the XC (Cruz et al., 2015).” Paragraph 7:

**C16:** In my opinion, the HGT debate has been put to bed by the thorough work in Maeda et al. 20219 but is missing from your discussion of this topic.

**R16:** the last part of the text has been modified to avoid overinterpretation of data and to provide a better fit between our conclusions and the experimental set-up. The part about HGT has been removed.

# Reviewer report: Reviewer response for version 1

**DOI:** 10.21956/openreseurope.17448.r33325

**Published:** 2023-07-19

**Authors:** Yoichi Yusa

**Affiliations:** 1 Faculty of Science, Nara Women's University, Nara, Nara Prefecture, Japan

This is an interesting study reporting the different performance of the sacoglossan sea slug *Elysia viridis* that fed on two algal species. Overall, I feel that the manuscript is well written and scientifically sound. Thus, I have only a few comments.

**Minor comments:**

1. In the introduction, similar studies in which a sea slug is fed two different algae are not referred to. I think that such references are needed to make the originality of your study clearer (as you did in the discussion).
2. Figures: Orange and blue colors with different degrees of paleness are difficult to see. I suggest the authors to use clearer patterns.
3. P.2, paragraph 1, 5 lines from the bottom: Italicize “Chaetomorpha”.
4. P.2, paragraph 2, 4 lines from the bottom: Although it may sometimes be considered an example of endosymbiosis, it is not, in my opinion, because the chloroplast is not an organism as you wrote.
5. P.7, in the middle of paragraph 3: “hindering their performance once integrated in the host cells”. Please clarify that the “host” is the sacoglossan here.
6. Figure 4 legend: Use the bold letters to the end of the first line.

Is the study design appropriate and does the work have academic merit?

Yes

Is the work clearly and accurately presented and does it cite the current literature?

Yes

If applicable, is the statistical analysis and its interpretation appropriate?

Yes

Are all the source data underlying the results available to ensure full reproducibility?

Yes

Are the conclusions drawn adequately supported by the results?

Yes

Are sufficient details of methods and analysis provided to allow replication by others?

Yes

Reviewer Expertise:

Aquatic ecology

I confirm that I have read this submission and believe that I have an appropriate level of expertise to confirm that it is of an acceptable scientific standard.

# Response

**Published:** 2023-07-29

**Authors:** Luca Morelli

Dear Dr. Yusa,  We kindly appreciate your comments on the manuscript. We will start now to elaborate a version including your changes and suggestions. 

 Best regards Luca

# Response

**Published:** 2024-02-17

**Authors:** Luca Morelli

R1 Yoichi Yusa **C1**: This is an interesting study reporting the different performance of the sacoglossan sea slug Elysia viridis that fed on two algal species. Overall, I feel that the manuscript is well written and scientifically sound. Thus, I have only a few comments.

**R**: We thank the reviewer for the positive feedback and for pointing out some mistakes in the manuscript. In the new version, we have addressed and corrected them.  

 Minor comments: In the introduction, similar studies in which a sea slug is fed two different algae are not referred to. I think that such references are needed to make the originality of your study clearer (as you did in the discussion).

**R:** We have included reference to studies comparing photosynthetic sea slugs fed with specific algae. It reads, “Experimental studies on polyphagous sea slugs fed with specific algal chloroplast donors show that functionality and longevity of kleptoplasts depend on plastid origin (Curtis et al. 2015; Cartaxana et al. 2023).”  

 Figures: Orange and blue colors with different degrees of paleness are difficult to see. I suggest the authors to use clearer patterns.

**R:** Different degrees of paleness are not used in the figures. We opted for solid versus pattern fill for the comparison of alga and sea slug (fed with that specific alga) and different colours (blue versus yellow) for the comparison of different sea slugs (or different algae).  

 P.2, paragraph 1, 5 lines from the bottom: Italicize “Chaetomorpha”.

**R:** Corrected.  

 P.2, paragraph 2, 4 lines from the bottom: Although it may sometimes be considered an example of endosymbiosis, it is not, in my opinion, because the chloroplast is not an organism as you wrote.

**R:**  We agree with the reviewer, but the fact is that it is sometimes referred in the literature as endosymbiosis, although, as referred in the sentence, the chloroplast does not constitute an organism.  

 P.7, in the middle of paragraph 3: “hindering their performance once integrated in the host cells”. Please clarify that the “host” is the sacoglossan here.  

**R:** Clarified. It reads, “hindering their performance once integrated in the animal host cells.”  

 Figure 4 legend: Use the bold letters to the end of the first line.

**R: **Corrected.
